# Glioblastoma and Melatonin’s Effects: A Narrative Review

**DOI:** 10.3390/cancers18040703

**Published:** 2026-02-21

**Authors:** Gaia Favero, Francesca Sulas, Mauro Labanca, Francesco Scilla, Corrado Federico Punzi, Claudio Lonati, Rita Rezzani

**Affiliations:** 1 Department of Clinical and Experimental Sciences, Università degli Studi di Brescia, 25123 Brescia, Italy; gaia.favero@unibs.it (G.F.); francesca.sulas@unibs.it (F.S.); mauro@maurolabanca.com (M.L.); corrado.f.punzi@gmail.com (C.F.P.); claudio.lonati@unibs.it (C.L.); 2 Interdepartmental University Center of Research Adaption and Regeneration of Tissues and Organs-(ARTO), Università degli Studi di Brescia, 25123 Brescia, Italy; 3 Italian Society for the Study of Orofacial Pain (Società Italiana Studio Dolore Orofacciale—SISDO), 25123 Brescia, Italy; 4 Department of Head, Neck and Sense Organs, School of Dentistry, Catholic University of Sacred Heart, Fondazione Policlinico Universitario “A. Gemelli”—IRCCS Rome, 00135 Rome, Italy; francesco.scilla@unicatt.it

**Keywords:** glioblastoma, melatonin, adjuvant treatments

## Abstract

Glioblastoma (GB) is an extremely aggressive, highly invasive brain tumor of astrocytic or oligodendrocyte glial origin. This tumor often infiltrates adjacent healthy brain tissue and can migrate significant distances from the primary tumor site. Given the poor overall survival of GB patients and the limited efficacy of current local and systemic treatments, new therapeutic strategies are needed to improve outcomes, reduce side effects, and enhance patients’ quality of life. In recent years, the potential chemotherapeutic effects of natural molecules have been investigated, either as primary agents or in combination with established chemotherapies in various types of cancer. Melatonin (MLT, N-acetyl-5-methoxytryptamine) is an endogenous indolamine primarily secreted by the pineal gland. MLT appears to discriminate between normal and tumoral cellular contexts and to modulate appropriate actions, thereby acting as a “smart killer” against cancer cells; however, further studies are needed to clarify this apparently paradoxical behavior. This review aims to summarize recent findings on the potential regulatory role of MLT in the modulation of key intracellular pathways in GB, underlining its potential role as a complementary adjuvant to conventional therapies. The clinical advantage of MLT in GB patients has not been demonstrated in randomized controlled trials; therefore, multicenter studies with well-defined objectives, appropriate dosage schedules, and clear patient classification are needed.

## Simple Summary

Glioblastoma is one of the most aggressive and difficult-to-treat brain tumors, with patient survival remaining very limited despite advances in chemotherapy, surgery, and radiotherapy. For this reason, new strategies are needed to improve treatment effectiveness and reduce side effects. Melatonin is a natural molecule produced by the pineal gland, best known for regulating sleep, but growing evidence suggests that it may also have antioxidant and anticancer properties. This review summarizes current knowledge on glioblastoma and examines recent studies showing how melatonin can influence the cellular mechanisms involved in tumor growth and progression. Overall, melatonin appears to be a promising complementary agent to conventional treatments and may offer new perspectives for future research in tumor management. To date, however, the data are fragmentary and unclear; several experimental studies do not demonstrate clinical efficacy, and only a few clinical studies are available.

## 1. Introduction

According to the 2021 World Health Organization (WHO) guidelines [1], gliomas are classified by invasiveness into diffuse and circumscribed types and grades 2, 3, and 4. Grade 4 diffuse gliomas are synonymous with glioblastoma (GB) multiforme [2]. The definition “multiforme” is not a well-defined characteristic since these tumors do not have a single and constant morphological feature and are not linked to a particular cell type but to the total alteration of the tissue [3]. For these reasons, we will use the term GB to define this type of tumor. GB accounts for ~50% of all malignant central nervous system (CNS) tumors [4,5] and is characterized by rapid growth, high invasiveness, and poor survival outcomes, with only modest improvements over the past 30 years [6,7,8]. According to the Cancer Genome Atlas project, GB activates several signaling and metabolic pathways critical for tumor cell survival and proliferation [9,10]. Tumor progression is strongly influenced by the tumor microenvironment, particularly a proinflammatory state that disrupts redox homeostasis and promotes gliomagenesis [10,11].

Current standard treatment is based on surgical resection followed by chemotherapy, radiotherapy, and stem cell transplantation [4]. Despite the use of aggressive multimodal therapeutic approaches and continuous medical advances, the median survival of patients remains low [4]. Indeed, current treatment strategies often fail to significantly improve long-term survival, highlighting the urgent need for novel therapeutic approaches [12].

For this reason, increasing attention has been paid to adjuvant and alternative treatments, particularly antioxidant strategies aimed at modulating oxidative stress and reducing chemotherapy-related toxicity [13,14,15]. Several antioxidants, including glutathione (GSH), superoxide dismutase (SOD), catalase (CAT), carotenoids, flavonoids, and polyphenols, have shown efficacy in GB models [16]. Among these, melatonin (MLT; N-acetyl-5-methoxytryptamine) has emerged as a particularly interesting candidate due to its broad biological activity and its ability to modulate multiple molecular pathways involved in cellular metabolism and homeostasis [17].

Although evidence suggests MLT’s potential role in cancer, several critical aspects regarding the mechanisms underlying its antitumor effects remain unclear. A deeper understanding of the pathways through which MLT influences tumor metabolism may support the development of new therapeutic strategies aimed at improving both survival and quality of life for GB patients in the context of preventive, predictive, and personalized medicine. Given the remarkable pleiotropy of MLT [18,19,20], multiple and interconnected mechanisms of action are likely involved.

This review summarizes and examines recent in vitro and in vivo studies evaluating MLT alone or in combination with conventional chemotherapeutic agents in GB, hoping to provide a framework for the rational use of MLT in GB therapy. We examined preclinical evidence supporting potential synergistic effects between MLT and standard GB therapies. Finally, based on encouraging preclinical findings, we highlight the need for further studies to better clarify the antitumor properties of MLT and to support its potential translation into clinical practice.

## 2. Melatonin in Tumor Protection: Mechanisms and Molecular Markers

MLT is an indolamine involved in regulating multiple physiological processes, particularly the circadian rhythm. It is primarily secreted by the pineal gland during the night but is also synthesized in other tissues, including the brain, skin, bone marrow, lymphocytes, retina, and gastrointestinal tract [21]. MLT synthesis begins with tryptophan and serotonin, and it is metabolized in the liver, primarily by cytochrome P450 enzymes.

MLT is a potent antioxidant and scavenges reactive oxygen species (ROS), thereby protecting cells from DNA damage and inhibiting tumor initiation [22]. Moreover, MLT induces up-regulation of antioxidant enzymes such as SOD, CAT, peroxidase, and GSH peroxidase [23].

Several studies have also demonstrated that MLT has anti-inflammatory effects [24,25], suppresses endothelial growth factor (EGF) [26] and hypoxia-inducible factor 1 (HIF-1) expression [27], and promotes apoptosis through mitochondrial dysfunction and caspase activation in neural cells [28,29].

Remarkably, MLT stimulates the functions of mitochondria, promoting their biogenesis, increasing adenosine triphosphate (ATP) production, and reducing free radical generation in tumor cells [21,30]. In addition, MLT regulates/modulates fission and fusion processes and prevents cell death through apoptosis [21].

Studies have demonstrated that MLT can limit the metabolism of tumor cells by inhibiting pyruvate dehydrogenase, reducing glycolysis, increasing oxidative stress, and inhibiting cancer cell growth [30,31].

### 2.1. Anticancer Action of Melatonin on Glioblastoma

It is important to note that there is no clear evidence from clinical trials that MLT alone can induce regression of GB neoplastic mass. However, it may be considered a possible adjuvant to address sleep dysregulation associated with cancer and to enhance sleep efficiency, with virtually no side effects [32,33]. Sleep disorders are debilitating symptoms also in brain tumor patients [33,34,35]. It is widely known that MLT is essential for the physiological regulation of the sleep cycle, and the disruption of this cycle negatively impacts the physical and mental health of patients, impairing daytime functioning [36,37]. Nevertheless, the relationship between sleep disorders, MLT and GB is multifaceted and intriguing. A possible target for sleep interventions is increasing MLT serum concentrations by MLT endogenous administration; however, further studies are needed to understand the mechanism of action. Reported side effects in short-term use include dizziness, headache, nausea, and sleepiness, which have been observed at levels comparable to placebo treatments [38,39].

Nevertheless, the increasing clinical use of supraphysiological doses warrants further investigation into the risks of both mild and serious adverse effects. At present, there is a lack of clinical trials with sufficiently large sample sizes to evaluate MLT’s mechanism(s) of action, route of administration, optimal dose, and potential adverse effects. Notably, exogenous oral doses of MLT greater than 0.3 mg already result in supraphysiological levels in humans [40], while the concentrations reached in relevant brain regions remain difficult to predict. MLT higher doses and prolongation of supraphysiological levels may promote the loss of its effectiveness on the sleep cycle, and with lower doses it can regain its effectiveness [41]. Consequently, both the optimal dose of MLT and the timing of its administration require further evaluation. Therefore, the use of an appropriate MLT dose, which best mimics its physiological circadian rhythm, will avoid prolonged, supraphysiological blood levels.

Further clinical studies are needed to better understand MLT’s mechanism of action against GB and to evaluate MLT as an antineoplastic therapy alone, not just as a supplement to more invasive therapies [42].

In the following paragraphs, MLT’s effects on GB from in vitro/in vivo studies and clinical trials are described.

### 2.2. Melatonin’s Effects on Glioblastoma: “In Vitro” and “In Vivo” Studies

Although several in vitro studies have demonstrated the antineoplastic activity of MLT against GB, evidence from in vivo animal models and clinical studies remains extremely limited. Translation of preclinical research into clinical application is limited by the lack of an adequate animal model of GB. The most appropriate transgenic rodent model of GB will be obtained through genetic engineering, which allows the selective introduction of mutations relevant to human GB that are suitable for preclinical testing of personalized therapies [43].

Table 1 summarizes the effects of MLT on GB growth demonstrated in in vitro studies conducted before 2021, while Table 2 reports the two clinical studies in humans published before that year. Notably, no in vivo animal studies were available prior to 2021.

To date, only two clinical trials have investigated the use of MLT, either alone or in combination with chemotherapy agents or radiotherapy, in patients with GB (Table 2). Furthermore, these studies are old and present several limitations, including a small number of enrolled patients, the use of supraphysiological doses of MLT, heterogeneous tumor histotypes, and a limited understanding of MLT’s mechanism(s) of action. For these reasons, these clinical trials avoid any implication of clinical efficacy.

Evidence emerging from 2021 to the present indicates that the evaluation of MLT, both as a single agent and, more importantly, in combination with conventional therapies, has attracted increasing scientific interest. For this reason, these studies are discussed in detail.

### 2.3. Melatonin in Glioblastoma Treatment: “In Vitro”, “In Vivo”, and Clinical Studies

**Figure 1:**
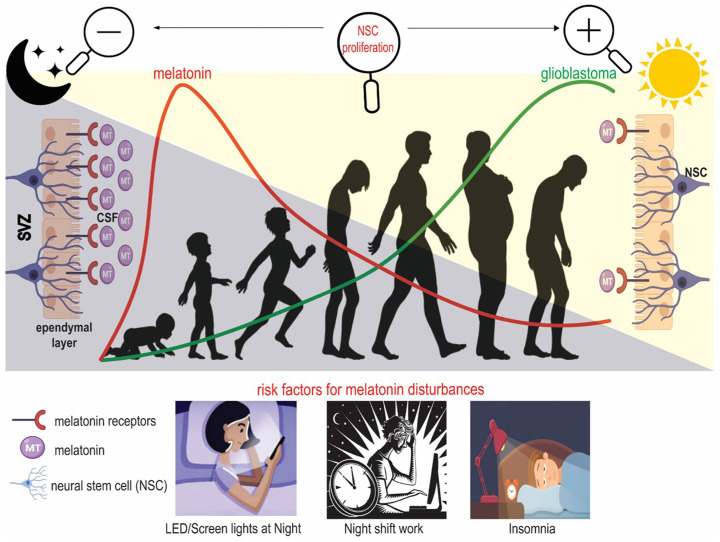
Melatonin and the incidence of glioblastoma. Children between 5 and 10 years old have maximum melatonin levels, which quickly decrease after this age. Melatonin is released in the third ventricle and reaches the cerebrospinal fluid (CSF). In the subventricular zone (SVZ), neural stem cells (NSCs) are in contact with melatonin in the CSF, which determines their fate. NSCs are under the control of melatonin synthesis; high levels of melatonin at night decrease NSC proliferation. Alterations in melatonin production may stimulate glioblastoma incidence. Illustration from Ghareghani et al. [59] (License number: 6183660495085).

Using in vivo imaging approaches, Ghareghani et al. [59] demonstrated that neural stem cells (NSCs), mainly present in the subventricular zone (SVZ) of the lateral ventricle, exhibit several mutations responsible for gliomagenesis and GB induction. The proliferation of these cells is regulated by MLT, and 70% of mitosis occurs during the day. Moreover, the authors demonstrated that stimulating MLT synthesis by keeping mice in constant darkness for 3–7 days resulted in a significant reduction in NSC division. In contrast, when they suppressed MLT synthesis by keeping the mice under continuous light for 3 or 7 days, the division of NSCs increased. During these experiments, the authors used luzindole, a non-selective inhibitor for MT1/MT2 receptors, and observed that cell division was significantly promoted. Furthermore, the possibility that the circadian rhythm and MLT synthesis control the proliferation of NSCs and that GB originates from the SVZ induced Ghareghani et al. [59] to suggest a correlation between MLT and GB. The mechanism explaining the association between MLT, circadian rhythm, and GB is described in Figure 1.

Fernandez-Gil et al. [60] studied metabolic processes (apoptosis, ROS production, ATP depletion, and pH microenvironment) in cell lines responsible for initiating glioma tumors. They used five glioma tumor cell lines derived from intraoperative tissue samples from GB patients (three males and two females aged between 59 and 63 years). These cell lines were treated at different time points with MLT (1.5 and 3.0 nM), and the effects on the tumor were evaluated. MLT reduced proliferation, apoptosis, and viability, and it had positive effects on mitochondria and glycolysis. MLT was most effective when administered at a high dose. Notably, GB, like other tumors, requires an alkaline microenvironment to survive. Fernandez-Gil et al. [60] demonstrated that MLT reverses the pH level in GB, resulting in the metabolic death of tumoral cells. It is important to note that reversing the microenvironment pH level induces acidosis, ATP depletion, and tumor cell death. MLT switches off glycolytic function in several tumors, causing a decrease in tumor growth and proliferation [61,62,63,64]. Moreover, Fernandez-Gil et al. [60] demonstrated that these effects are not exclusively linked to ROS production in mitochondria, as ROS levels are not necessarily generated there. The increase in ROS levels is explained by a decrease in nicotinamide adenine dinucleotide phosphate (NADPH) turnover into NAD following a reduction in lactate dehydrogenase A (LDHA) expression.

The reduction in MCT4 and LDHA could be the origin of the pH imbalance caused by MLT. However, other proton transporters, such as sodium/proton exchanger 1 (NHE1) or carbonic anhydrase IX (CAIX), that contribute to the movement of protons across the membrane should be considered in further research. Future rescue and loss-of-function assays are necessary to confirm the cause–effect of this correlation.

ROS levels decrease cell proliferation as well as inducing cell cycle arrest at G1 or G2/M; the mitosis phase requires alkaline pH, so G2 arrest supports the finding of an acidosis microenvironment [65]. Fernandez-Gil et al. [60] suggested that proton transporters, such as sodium/proton exchanger 1 (NHE1), be evaluated to better explain the cause–effect relationship of this association. Moreover, the authors stimulated this research, inviting others to explore new therapeutic frontiers in this direction. Then, Fernandez-Gil et al. [65] investigated the effects of MLT in vivo by injecting tumor cells into athymic mice and treating them intratumorally with MLT (3%) for 5 days a week for 2 weeks. Similar findings obtained in vitro were shown in vivo in these animal models.

As reported by Fernandez-Gil et al. [60], this study is the first to demonstrate the role of MLT in reversing the GB microenvironment. However, the study presents some limitations, and the authors appropriately propose directions for further research, including the use of intracranial models to evaluate local MLT delivery to the tumor and to decode the mechanisms underlying MLT-induced cytosolic acidosis. Furthermore, the GB animal model used (xenografted athymic nude mice) exhibited a short survival.

**Figure 2:**
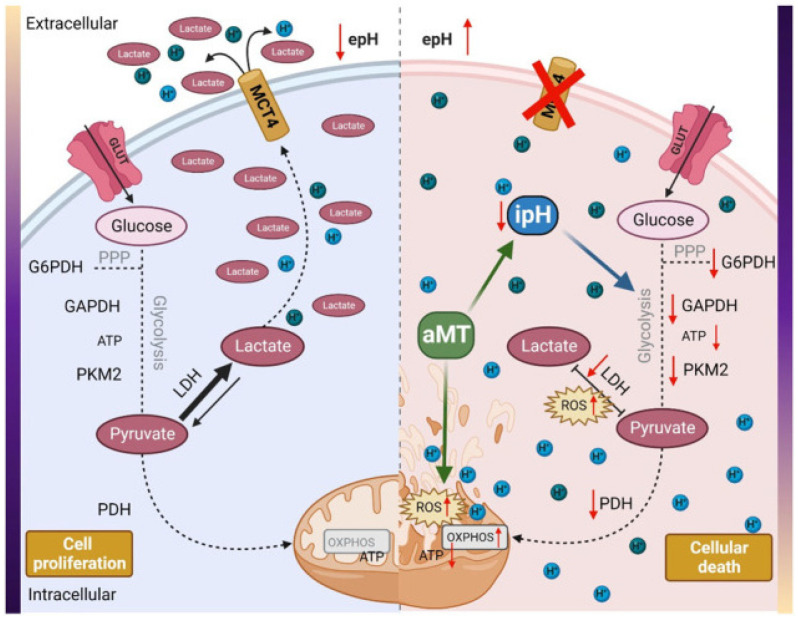
Effects of melatonin in glioblastoma. aMT, melatonin; ipH, intracellular pH; epH, extracellular pH; H+, proton; LDH, lactate dehydrogenase; ROS, reactive oxygen species; PKM2, pyruvate kinase; PDH, pyruvate dehydrogenase; GAPDH, glyceraldehyde-3-phosphate dehydrogenase; PPP, pentose phosphate pathway; G6PDH, glucose-6-phosphate dehydrogenase; GLUT, glucose transporters; MCT4, monocarboxylate transporter 4; ATP, adenosine triphosphate. Illustration from Fernandez-Gil et al. [60]. This article is an open-access article distributed under the terms and conditions of the Creative Commons Attribution (CC BY) license 4.0.

In this regard, the authors summarized the positive effects of MLT, suggesting that it inhibits the proliferation of GB, changes intracellular pH, and increases oxidative stress (Figure 2).

These results further support the role of the microenvironment, ROS, and NHE1 in MLT’s anti-tumor effects.

MLT also plays a role in the suppression of GB by modulating the expression of markers involved in the formation of the extracellular matrix (ECM). Recently, Olmedo-Moreno et al. [66] demonstrated that MLT enhances the antitumor effects of mesenchymal stromal cells (MSCs) on both in vitro and in vivo GB models. MSCs have been studied as a therapeutic strategy in cancer management because of their ability to remodel the extracellular matrix (ECM) and to prevent tumor progression.

In this regard, the authors showed that pretreating MSCs with MLT (25 μM) in vitro for 24 h in growth media leads to positive effects on ECM dynamics, inducing collagen deposition and stimulating the expression of proteins such as collagen 1A2 (COL1A2) and collagen 12A1 (COL12A1). These results indicated that increased expression of these proteins induced the formation of a collagen capsule around tumor cells, thereby limiting their invasion [67]. Similar findings were obtained in in vivo studies on experimental GB models (athymic nude mice).

A potential correlation between collagen and the survival of patients with GB has been demonstrated, as GB has no opportunity to expand and spread in the presence of collagen. However, the specific condition of the tumor environment is a major limiting aspect, together with the contradictory results reported when MSCs are used for cancer treatment.

Another finding suggests that modulating the expression of enzymes involved in MLT synthesis or altering the levels of MLT transporters can strengthen the effects of MLT, making it more effective in controlling tumor growth [68].

Finally, it is important to emphasize that stimulating the cellular mechanisms involved in MLT synthesis could offer an innovative therapeutic strategy for patients with GB, as reported by Olmedo-Moreno et al. [66]. However, the efficacy and safety of MLT treatment in MSCs in tumors must be verified by new experimental and clinical studies [66].

More recently, evidence has highlighted the positive effects of MLT in an in vivo animal model (male Wistar rats). GB was induced by the inoculation of C6 glioma cells. MLT (1.5 mg/kg body weight) was administered via orogastric gavage starting on day 17 of the experimental protocol, and treatment continued once daily until the end of the study. Animals were euthanized 28 days after assignment to the experimental groups. The authors demonstrated that MLT prevented malignant tumor progression by inhibiting the proliferation of GB-like stem cells and reducing tumor-associated vascularization associated with circadian rhythm alteration. Several angiogenesis-related markers were modulated, including vascular endothelial growth factor (VEGF), a key regulator of angiogenesis. These findings suggest that VEGF is an important mediator in reducing tumor growth, confirming the role of MLT in this context [26].

### 2.4. Melatonin Combined with Antineoplastic Drugs in Glioblastoma Treatment: “In Vitro”, “In Vivo”, and Clinical Studies

To date, a few studies have evaluated the effects of MLT in combination with antineoplastic drugs to confirm its role as an adjuvant in therapeutic strategies. These studies are listed below.

In 2021, Ertilav et al. [69] demonstrated that the combination of docetaxel (DT), MLT, and selenium (Se) in the treatment of GB in mice and SH-SY5Y cells decreased adverse peripheral oxidative neurotoxicity and pain. DT, one of the main antineoplastic agents, induces nociception and mitochondrial oxidative stress in the sciatic nerve and dorsal root ganglion (DRG). The authors studied the activation of transient receptor potential vanilloid 1 (TRPV1) channel, which is a member of the transient receptor potential (TRP) superfamily, in GB and cell tumor lines. The MLT and Se doses administered to mice were 10 mg/kg and 1.5 mg/kg, respectively; these antioxidants were used together with DT. The doses of these antioxidants in cultured cells were 1 nM for MLT, 1 μM for Se, and 10 nM for DT.

The results demonstrated that, when MLT was used in combination with Se, the effects were greater than when MLT was administered alone. In conclusion, they showed that MLT and Se reduced the side effects of DT by decreasing TRPV1 channel activation and supporting the thiol antioxidant redox system in the DRG and SH-SY5Y cells. In this context, the role of MLT concerns pain reduction rather than the inhibition of tumor proliferation.

Hernández-Cerón et al. [70] showed that the co-treatment of MLT (0.18–6 mM) and albendazole (0.16–1.25 μM) in the U87 human GB cell line and C6 and RG2 rat malignant glioma cell lines has similar effects reported above. The authors suggested that, compared with chemotherapy, the combined treatment induces significantly more apoptotic and autophagic pathways in these cell lines. The inhibitory effects of albendazole on cancer cell proliferation were amplified when it was combined with MLT. Thus, the authors justified these findings, suggesting that the combination of MLT and albendazole is an important strategy to improve the treatment of GB.

Wang et al. [33] demonstrated that the combined treatment of MLT and nimotuzumab, a humanized monoclonal antibody that targets the epidermal growth factor receptor (EGFR), increased cytotoxicity and apoptosis in human glioma cell lines. The human glioma (U118-MG, U87-MG, LN229, KNS42, GB1) cell lines and the primary patient-derived GB cell lines were treated with MLT and nimotuzumab at different doses (10 μM, 100 μM, 1000 μM); the optimal dose used was 100 μM. The results suggested that nimotuzumab interferes with the binding of EGF to the EGFR, and MLT inhibits EGFR activity by interacting with the tyrosine kinase present in the intracellular segment of the EGFR. MLT and nimotuzumab work in synergy, and they can be a dual-drug strategy targeting only one protein.

Similar experiments were also performed in tumor-bearing mice, which were treated with MLT (50 mg/kg per os) and nimotuzumab (10 mg/kg intravenous); the results showed that MLT enhances nimotuzumab capacity by regulating the EGFR pathway. The authors concluded that clinical studies need to consider individual patient factors such as age, other medical conditions, and treatments.

Recently, Tang et al. [71] reported the administration of temozolomide (TMZ; 25–100 μM) combined with MLT (500 μM) in human GB cell lines (U87-MG, U228-MG) and a murine glioma cell line (GL261). Notably, TMZ is frequently used together with radiotherapy as part of the first-line treatment of GB [72]. The results suggested that this combination of substances reduced GB cell proliferation, migration, and invasion. The processes involved in these effects are mediated through the NF-kB and cyclooxygenase-2 (COX-2) signaling pathway. MLT enhances the activity of TMZ by suppressing NF-kB activation and downregulating COX-2 expression. Moreover, MLT induces apoptosis by activating the caspase-3 pathway.

**Figure 3:**
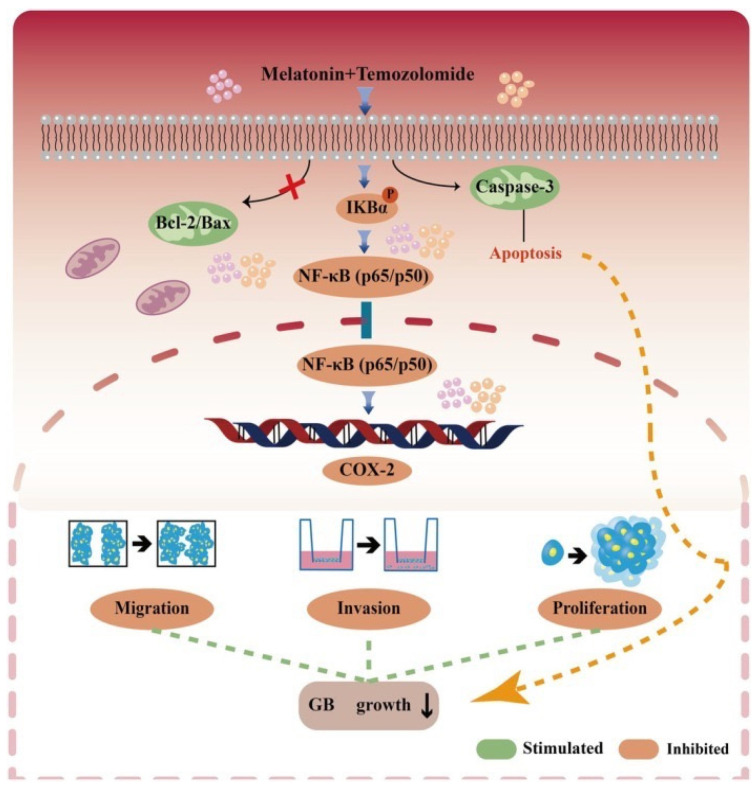
Main molecular pathways mediating melatonin effects on glioblastoma cells in combination with temozolomide. Bcl-2/Bax, B-cell lymphoma 2/Bcl-2-associated X protein; IKBα, inhibitor of kappa B-alpha; NF-kB, nuclear factor kappa-light-chain-enhancer of activated B cells; COX-2, cyclooxygenase-2; GB, glioblastoma. Illustration from Tang et al. [71]. This article is an open-access article distributed under the terms and conditions of the Creative Commons Attribution (CC BY) license 4.0.

These effects indicate that MLT is a promising strategy for reducing GB resistance to antineoplastic agents. The proposed mechanisms are reported in Figure 3.

Furthermore, Trejo-Solís et al. [73] reported synergic antitumor effects of MLT combined with different drugs in the treatment of breast, colorectal, prostate, gastric, thyroid, and pancreatic cancer, as well as GB. In this review, the authors supported the findings obtained by Sung et al. [46], showing the effects of the combined administration of MLT and vorinostat (a drug used to treat GB) both in vivo and in vitro.

The combination of MLT (1 mM) and vorinostat (8 μM) was evaluated in cell lines in vitro (GSC 267 and U87MG cells). The combination reduced the viability of both cell lines and induced apoptosis more efficiently than the use of MLT or the drug alone. The treatment stimulated poly-(ADP)-ribose polymerase and caspase-3 split; it also induced the activation of the transcription factor EB (TFEB) in GB and in cell lines such as U87. Moreover, in vivo treatment with MLT (15 mg/kg) and vorinostat (25 mg/kg) reduced tumor growth and increased median survival in GSC267 nude mice. No positive effects were observed with MLT or vorinostat alone. Thus, the scientists suggested that the combination of MLT and vorinostat inhibits tumorigenesis and resistance by modulating TFEB activation in GB.

The positive effects of MLT (100 mg/day), 5-methoxytryptamine (5-MTT), pinealine (PNL), and cannabidiol (CBD, 10 mg twice daily) in association with tumor resection and radiotherapy have been demonstrated by Lissoni et al. [74]. In particular, patients treated with chemotherapy and radiotherapy in combination with the neuroendocrine regimen and CBD exhibited significantly greater tumor regression and a higher five-year survival rate compared with patients treated with chemotherapy and radiotherapy alone.

This study confirmed that the concomitant administration of the natural molecules listed above with standard treatments enhances the efficacy of radiotherapy and chemotherapy, due to their inhibitory effects on brain tumor growth [75,76] and their immunostimulatory properties [77]. However, the results of this study are limited to the neuroimmune regimen employed and could be further enhanced by combining it with another important endogenous molecule, such as angiotensin-(1–7), which appears to exert antiproliferative and antiangiogenic effects [78,79].

Consistent with these findings, a case report described that combined treatment with cannabinoids, MLT, and ozone contributed to the complete recovery of a 36-year-old woman with GB [80]. Following partial tumor resection and radiotherapy, the patient refused standard chemotherapy because of intolerable side effects and started supportive treatment based on the above-mentioned agents. MLT was administered orally in combination with cannabinoids and oxygen–ozone (O_2_O_3_) therapy at an initial dose of 100 mg once daily, which was increased by 100 mg every four days up to a maximum dose of 2 g. The treatment consisted of cycles of three months on therapy followed by three months off. With the use of O_2_O_3_, MLT, and legal cannabis therapy, the GB patient completely recovered. However, as acknowledged by the authors themselves, there is still insufficient research to fully confirm these findings, and further well-designed clinical trials are certainly needed [80].

## 3. Challenges, Perspectives, and Conclusions

Current evidence indicates that effective GB treatment requires an integrated and personalized approach combining surgery, chemotherapy, and radiotherapy with molecular diagnostics and precise patient stratification. Challenges such as the blood–brain barrier, cellular resistance, and treatment costs remain key limitations. Although the biological effects of MLT in various pathological conditions have been known for many years, and several studies support its antitumor potential, its role in GB is still innovative and not yet adequately elucidated, as highlighted by Gurunathan et al. [81] and Alomari et al. [82]. A substantial disparity persists between promising preclinical findings and their clinical validation, underscoring the need for further studies to clarify MLT’s antitumor mechanisms and its potential clinical implications as an adjuvant therapy for GB and other tumors.

Recent evidence, mainly from 2021 onward, suggests that evaluating MLT alone or in combination with conventional therapies represents an emerging and relevant field that may contribute to improved GB management. However, current studies are often fragmented, focusing on isolated molecular mechanisms without exploring their interconnectivity or translational implications. Filling these gaps is crucial to support rational drug design and formulation, develop synergistic treatment strategies, and potentially improve clinical outcomes in a disease with limited effective options [82].

Unlike conventional treatments that primarily target single pathways, MLT’s broad activity profile suggests its potential to enhance standard therapies and mitigate resistance. However, the lack of clinical trials and the limited understanding of MLT’s dose, formulation, and mechanisms when combined with conventional treatments emphasize the need for well-designed translational and clinical studies.

## Figures and Tables

**Table 1:** In vitro studies investigating the effects of melatonin in glioblastoma. GB, glioblastoma; MLT, melatonin; TMZ, temozolomide; NSC, neural stem cell.

**Study**	**In Vitro Model**	**MLT Dose**	**Outcome**
Zhou et al. [44]	A172 and U87-MG GB cells.	0, 0.1, 0.2, 1, and 2 mM for 24 h	MLT induced autophagy in a concentration-dependent manner.
Lai et al. [45]	Human glioma cells (U251), human glioma cells (U87), human glioma cells (A172), mouse glioma cells (ALTS1C1), and human monocytes (THP-1).	0, 0.25, 0.5, 1, or 3 mM for 24 h	MLT inhibited IL-1β-induced ICAM-1 and VCAM-1 expression and upregulated SIRT1.
Sung et al. [46]	Human 293T, A172, and U87MG cells.	0–0.5 mM for 24 h	MLT dose-dependently reduced transcription factor EB protein levels in U87MG cells, and the proliferation and viability of U87MG.
McConnell et al. [47]	Human GB cell line (U87-MG) and human GB primary cell line (MU1454).	1 mM, 1 μM, and 50 nM for 72 h	MLT decreased cell proliferation as a solo agent. As a pretreatment to TMZ, MLT successfully reduced cellular proliferation in both cellular lines by about 55–65%, compared to the controls. A reduction in proliferation was also observed in tumor stem-like cells.
Franco et al. [48]	Human glioma cell line U87MG.	1 mM and 3 mM for 72 h	MLT reduced the expression of several transcription factors that are associated with mitochondrial activity and upregulated in different types of cancer. MLT also induced mitochondrial membrane depolarization and apoptosis. The addition of MLT to TMZ increased the inhibitory effect on cell viability and proliferation.
Pan et al. [49]	Human U251 GB cells and TMZ-resistant U251 cell line (U251-TMZ).	0.1, 0.5, 1 and 2 mM for 12, 24, and 48 h	MLT reduced Nrf2 expression and survival rate in U251-TMZ cells, accompanied by increased reactive oxygen species levels.
Zheng et al. [50]	GB cell lines (U251 and T98G).	0.1–1000 μM for 10 days	MLT inhibited the viability of GB stem-like cells in a dose-dependent manner.
Chen et al. [51]	Tumor cells derived from GB patient specimens.	1 M for 5 days	MLT decreased GB stem cells’ self-renewal and clonogenic capability.
Martín et al. [52]	Neurospheroid cultures established from acute cell dissociation of human GB postsurgical specimens and human normal neural stem cells hNSC.100.	1–1000 μM for 1–7 days	MLT completely blocked cell proliferation at the highest dose and decreased self-renewal capability.
Zhang et al. [53]	Human U251 and U87 GB cell lines.	1 nM and 1 mM for 12–24 h	MLT blocked the migratory activities of both U251 and U87 GB cells under hypoxic stimuli.
González et al. [54]	Rat glioma cells (C6).	1 nM, 1 µM and 1 mM for 24 h	MLT induced a significant inhibition of steroid sulfatase activity and its mRNA expression.
González et al. [55]	Rat glioma cells (C6).	1 mM, 10 µM, 1 µM, 100 nM, 1 nM, and 10 pM for 3–4 days	The simultaneous addition of 17β-oestradiol and high dose of MLT resulted in a significantly lower cell proliferation than that of the 17β-oestradiol-treated cells and control (untreated) cells. MLT alone significantly decreased the aromatase activity of C6 cells.
An et al. [56]	SKNSH and U251 cell lines.	10 µM	MLT can significantly protect cells from apoptosis induced by H_2_O_2_ and amyloid beta-protein.

**Table 2:** Clinical trials investigating the effects of melatonin in patients with glioblastoma. GB, glioblastoma; MLT, melatonin.

**Study**	**Study Design**	**Participants**	**Outcome**
Lissoni et al. [57]	Patients were treated with MLT alone (20 mg/day in the dark period) or MLT plus Aloe vera tincture (1 mL twice/day)	50 patients suffering from lung cancer, gastrointestinal tract tumors, breast cancer, or brain GB	MLT plus Aloe vera extracts produced some therapeutic benefits, at least in terms of stabilization of disease and survival
Lissoni et al. [58]	Patients were randomized to receive radiotherapy alone (60 Gy) or radiotherapy plus MLT (20 mg/day orally)	30 patients with GB treated with radiotherapy	The percentage of survival at 1 year was significantly higher in patients treated with radiotherapy plus MLT than in those receiving radiotherapy alone

## Data Availability

No new data were created or analyzed in this study. Data sharing is not applicable to this article.
